# Supplementary material for: Frustrated self-assembly of non-Euclidean crystals of nanoparticles
Source: Nat Commun. 2021 Aug 13;12:4925. doi: 10.1038/s41467-021-25139-9 (PMC8363672; doi:10.1038/s41467-021-25139-9)
Supplement: Supplementary file 1 — Supplementary Information [file 41467_2021_25139_MOESM1_ESM.pdf]

Supplementary Information: Frustrated Self-Assembly of Non-Euclidean Crystals of Nanoparticles

Francesco Serafin,[1] Jun Lu,[2] Nicholas Kotov,[2] Kai Sun,[1] and Xiaoming Mao[1,*]
[1]*Department of Physics, University of Michigan, Ann Arbor, MI 48109-1040, USA*
[2]*Department of Chemical Engineering, University of Michigan, Ann Arbor, MI 48109, USA*
(Dated: July 10, 2021)

I. THE 600-CELL STRUCTURE

In this appendix we discuss the structure of the 600-cell, which is a "regular honeycomb" (i.e., a space-filling packing of polyhedra) of tetrahedra on the 3-sphere S^3.

Regular honeycombs can be classified by the Shläfli notation $\{p, q, r\}$ where q is the number of $p-$sided regular polygonal faces around a polyhedron's vertex, and r is the number of polyhedra around an edge. Calling Θ the dihedral angle of a polyhedron, when $r\Theta < (>) 2\pi$, the honeycomb has positive (negative) Gaussian curvature.

In this notation the 600-cell can be written as a $\{3, 3, 5\}$ tiling. It contains $N_0 = 120$ vertices, $N_2 = 720$ edges, $N_3 = 1200$ faces and $N_3 = 600$ tetrahedral cells.

Introducing Cartesian coordinates X^μ ($\mu = 1, ..., 4$) in 4D Euclidean space $\mathbb{E}^4$ (where the 3-sphere is embedded) and choosing the origin at the center of the 3-sphere, the vertices of the 600-cell belong to the hypersurface

$$(X^1)^2 + (X^2)^2 + (X^3)^2 + (X^4)^2 = R^2. \quad (1)$$

The radius R of the circumscribed sphere S^3 is related to the edge length of the tetrahedra a via

$$R = \phi\, a, \quad (2)$$

where $\phi = (1 + \sqrt{5})/2$ is the Golden Ratio.

The 600-cell can be organized in 20 close-packed tetrahelices measuring 30 tetrahedra in length [1]. The symmetry group of the 600-cell organizes these tetrahelices on the *icosahedron base*, where 12 fiber rings of length $10a$ (decagons) formed by shared edges of the tetrahelices are associated to the 12 vertices of the icosahedron base, forming a discrete Hopf fibration (Fig. 1). These rings are linked pairwise with Hopf link 1. At the same time, each of the 20 tetrahelices corresponds to each of the 20 triangular faces of the base icosahedron.

The discrete Hopf fibration by an icosahedron and a decagon is a useful tool to visualize the packing of tetrahelices in the 600-cell (Fig. 2 in the main text). For illustrative purposes, we align the icosahedron's 5-fold symmetry axis along the $z-$direction. Let γ be the polar angle: $\gamma = 0, \pi$ are the North and South poles of the icosahedron and the 10 remaining vertices belong to two latitudes at the North and South Tropics

$$\gamma_{\rm N,S} = \frac{\pi}{2} \mp \arctan\frac{1}{2}. \quad (3)$$

* maox@umich.edu

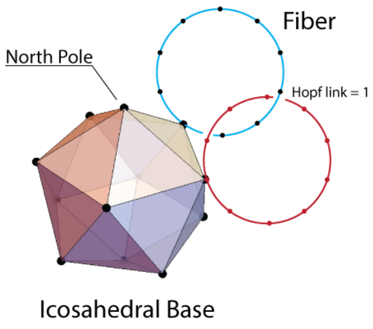

FIG. 1. The discrete Hopf fibration of the 600-cell. The base space is an icosahedron, and the fibers are non-intersecting decagons.

We start from $\gamma = 0$ and describe the order of the tetrahelices as we travel from the North to the South Pole in the base icosahedron. At $\gamma = 0$ there is a chain of 10 vertices, shared between 5 tetrahelices (one for each triangle on the base between $\gamma = 0$ and $\gamma = \gamma_{\rm N}$). This means that close to the North pole, there are 5 tetrahelices bundled perfectly around a common long edge. The North pole maps to the axis of the bundle, while the 5 vertices at $\gamma_{\rm N}$ map to the exposed corners of the 5 tetrahelices. The bundle has the topology of a solid torus, because the tetrahelices are closed in rings of 10 tetrahedra. The 5 edges at $\gamma_{\rm N}$ map to the outer surface of the torus, which contains $20 \times 5 = 100$ triangular tiles.

For $\gamma_{\rm N} < \gamma \leq \gamma_{\rm S}$, we encounter 10 tetrahelices arranged into a solid torus that wraps around the "polar bundle" (Fig. 2gh in the main text). In fact, there are 5 tetrahelices that coat the outer surface of the polar bundle, thus making a star-shaped torus with a cylindrical cavity inside and a zig-zag outer surface. The other 5 fit into the zig-zag surface thus completing the solid hollow torus. The surface exposed to the South pole has again 100 triangular tiles. The South pole bundle is identical to the one at the North pole, and wraps around the hollow torus, covering its exposed surface. The 600-cell is thus made of 3 nested solid tori, separated by 2 surfaces at $\gamma_{\rm N,S}$. Importantly, there are two ways of constructing the discrete Hopf fibration, depending on whether the tetrahelices wrap around each other with a right-handed or a left-handed rotation.

Following this discrete Hopf fibration, a continuous

polar-coordinate $\Theta^\mu = (\alpha, \beta, \theta)$ can be introduced

$$X^1 = R\cos\alpha\cos(\theta - \beta) \tag{4a}$$
$$X^2 = R\cos\alpha\sin(\theta - \beta) \tag{4b}$$
$$X^3 = R\sin\alpha\cos(\theta + \beta) \tag{4c}$$
$$X^4 = R\sin\alpha\sin(\theta + \beta) \tag{4d}$$

where X^ν are Cartesian coordinates of $\mathbb{E}^4$, and $\alpha \in [0, \pi/2]$ and $\beta \pm \theta \in [0, 2\pi[$ are angles on S^3. In this coordinate α correspond to the toroidal shells, and β and θ are aligned with the LH and RH tetrahelices.

The coordinates Eq. (4) are related to the Hopf fibration in a simple way. The icosahedral base mentioned in section I becomes the Base 2D sphere S^2 of the Hopf fibration with latitude-longitude coordinates $(\gamma = 2\alpha, \beta)$. The decagonal fibers become 1D circles S^1 parameterized by θ.

Using this coordinate, the 120 vertices of the 600-cell are at:

1. The north pole of the base icosahedron generates a fiber of 10 vertices
$$\alpha = 0, \quad \theta = \frac{2\pi k}{10}, \tag{5}$$
where $k = 0, ..., 9$.

2. The north tropical latitude of the base icosahedron generates 50 vertices lying on a surface
$$\alpha = \frac{\pi}{4} - \frac{1}{2}\arctan\frac{1}{2},\ \beta = \frac{2\pi m}{5},\ \theta = \frac{2\pi k}{10} + \frac{\pi}{10}, \tag{6}$$
where $m = 0, ..., 4$ and $k = 0, ..., 9$.

3. The south tropical latitude of the base icosahedron generates 50 vertices lying on a surface
$$\alpha = \frac{\pi}{4} + \frac{1}{2}\arctan\frac{1}{2},\ \beta = \frac{2\pi m}{5} + \pi,\ \theta = \frac{2\pi k}{10} + \frac{\pi}{10}, \tag{7}$$
where $m = 0, ..., 4$ and $k = 0, ..., 9$.

4. The south pole of the base icosahedron generates a fiber 10 vertices
$$\alpha = \frac{\pi}{2} \quad , \quad \theta = \frac{2\pi k}{10}, \tag{8}$$
where $k = 0, ..., 9$.

II. THE REFERENCE METRIC FROM THE 600-CELL

In this section we derive the reference metric $\bar{\mathbf{g}}$ of the ideal packing of tetrahedra from the 600-cell.

The 4D tangent vectors to S^3 associate with the coordinate $\Theta^\mu = (\alpha, \beta, \gamma)$ are

$$\mathbf{t}_\mu = \frac{1}{R}\frac{\partial \mathbf{X}(\Theta)}{\partial \Theta^\mu} \equiv R^{-1}\partial_\mu \mathbf{X}(\Theta) \tag{9}$$

where the factor of R^{-2} makes the tangent vectors dimensionless. The components of the metric tensor in the coordinates Eq. (4) are $\bar{g}_{\mu\nu} = \mathbf{t}_\mu \cdot \mathbf{t}_\nu$,

$$\bar{g}_{\mu\nu} = R^{-2}\partial_\mu \mathbf{X} \cdot \partial_\nu \mathbf{X} = \begin{pmatrix} 1 & 0 & 0 \\ 0 & 1 & -\cos 2\alpha \\ 0 & -\cos 2\alpha & 1 \end{pmatrix}. \tag{10}$$

The metric is block-diagonal ($\bar{g}_{\alpha\beta} = \bar{g}_{\alpha\theta} = 0$) so the $\alpha-$axis is everywhere orthogonal to the $\beta-$ and $\theta-$axis. Notice that $\bar{g}_{ij}$ depends only on the coordinate α. Fixing α =const gives a 2D toroidal surface in S^3. The metric is *constant* on this surface. The line element is

$$\begin{aligned} \mathrm{d}\bar{s}^2 &= \bar{g}_{\mu\nu}\mathrm{d}x^\mu \mathrm{d}x^\nu = R^2 \bar{g}_{\mu\nu}\mathrm{d}\Theta^\mu \mathrm{d}\Theta^\nu \\ &= R^2[\mathrm{d}\alpha^2 + \mathrm{d}\theta^2 - 2\cos 2\alpha\, \mathrm{d}\theta \mathrm{d}\beta + \mathrm{d}\beta^2], \end{aligned} \tag{11}$$

which can also be written as

$$\mathrm{d}\bar{s}^2 = R^2[\mathrm{d}\alpha^2 + \bar{g}_{ij}(\alpha)\mathrm{d}\Theta^i \mathrm{d}\Theta^j], \tag{12}$$

where $\Theta^i = (\beta, \theta)$. The 2-dimensional metric induced on the toroidal surfaces at fixed α is

$$\bar{g}_{ij}(\alpha) = \begin{pmatrix} 1 & -\cos 2\alpha \\ -\cos 2\alpha & 1 \end{pmatrix}, \tag{13}$$

and it is constant once we fix α. We recall that we inscribed the icosahedral base of the 600-cell in the base 2-sphere, and 2α is the latitude on the base S^2. We will use 2α to label both the latitudes of the icosahedral base and of the continuous S^2 base. For $\alpha_{N,S} = \pi/4 \mp 1/2\arctan(1/2)$ on the icosahedral base, we find two groups of 50 vertices arranged into a grid formed by the great circles along β and θ (see Fig. 2). The angle between the red and black lines is $\arccos(1/\sqrt{5})$. The surfaces $\Sigma_{\alpha_{N,S}}$ are separated by an angular distance $\Delta\alpha = 2(\alpha_S - \alpha_N)$. The surface $\alpha = \pi/4$ which is equidistant from $\Sigma_{\alpha_{N,S}}$ is the Clifford torus and does not contain any vertex of the 600-cell. The coordinate grid (β, θ) is orthogonal on the surface of the Clifford torus (see Fig. 2). The Clifford torus is the mid-surface of a shell whose upper and lower boundaries are $\Sigma_{\alpha_{N,S}}$.

The determinant of $\bar{g}$ is $\det \bar{g} = \sin^2 2\alpha$ and the reference volume element is

$$\mathrm{d}V = R^3\sqrt{\det \bar{g}}\, \mathrm{d}^3\Theta = (R^3 \sin 2\alpha)\mathrm{d}\alpha \mathrm{d}\beta \mathrm{d}\theta \tag{14}$$

The determinant of $\bar{g}$ vanishes at $\alpha = 0, \pi/2$ so the coordinate system (α, β, θ) is singular at the North and South pole of the base icosahedron. The singularities are analogous to the spherical coordinate system on the 2-dimensional sphere S^2: at the North and South pole the tangent vectors $\mathbf{t}_\beta$ and $\mathbf{t}_\theta$ are parallel. In fact, at $\alpha = 0$ the coordinates parametrize a 1-dimensional circle $\mathbf{X} = R(\cos\theta, \sin\theta, 0, 0)$ rather than a 2D surface. At the South Pole ($\alpha = \pi/2$), the embedding becomes $\mathbf{X} = R(0, 0, \cos(\theta + \beta), \sin(\theta + \beta))$ that is again a great circle $\mathcal{C}_{\pi/2}$ covered twice by $\beta + \theta \in [0, 4\pi]$. We fixed $\beta = 0$ to avoid the double-covering of $\mathcal{C}_{\pi/2}$.

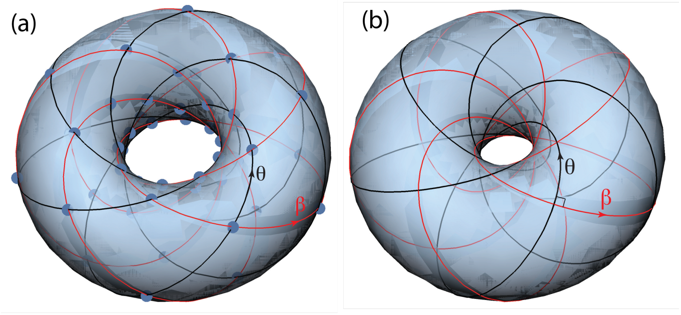

FIG. 2. (a) Group of 50 vertices (blue dots) on the toroidal surfaces $\Sigma_{\alpha_{N,S}}$ located at $\alpha_{N,S} = \pi/4 \mp 1/2 \arctan(1/2)$ (stereographic projection in $\mathbb{E}^3$). The angle between the red (β) and black (θ) curvilinear axis is $\arccos(1/\sqrt{5})$. Sterographic projections are conformal (angles are preserved by the projection) so the angles visualized in this figure are the same as we would see them inside S^3. The distances between nodes in the grid are distorted by the projection. (b) The Clifford torus ($\alpha = \pi/4$) (stereographic projection in $\mathbb{E}^3$). The coordinate lines (β, θ) are orthogonal here. There are no vertices of the 600-cell on the Clifford torus.

Figure 2 shows the grid formed by the curvilinear axis θ (black lines) and θ (red lines) on the surface of a torus. In order to draw the tetrahelices, we should connect these vertices with the ones on the neighboring toroidal shell. The tetrahelices that are aligned with the red lines are R, and those that are aligned with the black lines are L. Thus the 600-cell is composed by two bundles of tetrahelices linked pairwise: one contains only RH helices, the other only LH helices (see e.g. Fig. 2f in the main text).

It is worth noting that the 600-cell of flat tetrahedra is faceted: the interior of the tetrahedra is flat, while edges carry positive Gaussian curvature. The coordinate we introduced above is a coordinate of the 3-sphere S^3 that circumscribes the 600-cell. This is analogous to using the spherical coordinate of the 2-sphere for the circumscribed icosahedron: all vertices lie on the 2-sphere, but the interior of the triangles are flat and not on the 2-sphere. As an elasticity theory we build here for the self-assembly problem, this continuous coordinate ignores the flatness of the tetrahedra themselves, and approximate the geometric frustration by a homogeneous curvature. We view this continuous theory as an approximation of the actual material, where hard tetrahedra are connected by soft ligands. By using this continuum theory, we homogenize the discrete materials into a continuous media, where the hard tetrahedra plus the ligands are approximated as *spherical* tetrahedra.

This approximation is increasingly accurate in the limit of $a/R \to 0$ where a is the edge length of the tetrahedra and R is the radius of the 3-sphere. Although the crystalline 600-cell is not in this limit ($a/R = \phi^{-1} \sim 0.618$), we expect a continuous distribution of disclinations which greatly reduce this ratio. Sadoc showed that it is possible to preserve the topology of the fiber bundle and elongate the fibers at the same time, if one chooses specific disclinations that change the icosahedral base into a non-regular polyhedron [1]. Since the $\alpha-$helices are less dense than a tetrahedral self-assembly, in [1] the tetrahelices were not closely packed. The radius of S^3 effectively increases by introducing a density of defects, so the curvature of the non-Euclidean crystal proportional to $1/R^2$ decreases, making it more compatible with flat space.

III. THIN SHELL EXPANSION OF THE ELASTIC ENERGY

As we discussed in the main text, the geometric frustration of the elastic energy leads to stresses as the crystal grows in the 3D Euclidean space. This suggests that self-assembly favors thin sheets over thick bulk structures, which balances the surface tension and the elastic energy.

In order to find the actual configuration of a thin assembly, we must minimize the elastic energy over a thin region $\bar{M}$ of S^3. As discussed in the main text, we select a toroidal surface $\mathbf{X}_0(\beta,\theta) \equiv \mathbf{X}(\alpha_0,\beta,\theta)$ at a fixed $\alpha = \alpha_0$, and construct a sequence of toroidal surfaces around it varying $\alpha \in [\alpha_0 - h/(2R), \alpha_0 + h/(2R)]$. This region is the reference configuration of a 3D self-assembly of dimensions $h \times L \times W$. When the scales $h \ll W, L$ are well separated, we call it a thin sheet. Although the coordinates $\beta, \theta \in [0, 2\pi]$ in S^3, they are not bounded by these limits in this sheet, as they now have the topology of an open sheet, not a torus, as we discussed in the main text.

The material is not uniform across its thickness, so it is analogous to an elastic *thin shell* rather than a thin plate [2]. We define the thickness parameter

$$t \equiv R(\alpha - \alpha_0) \tag{15}$$

with dimensions of length, and expand $\bar{g}_{ij}(\alpha)$ around $\alpha = \alpha_0$

$$\bar{g}_{ij}(t) = \bar{a}_{ij} - 2t\bar{b}_{ij} + t^2\bar{c}_{ij} + o(t^3) \tag{16}$$

The constant tensors $\bar{a}_{ij}$ and $\bar{b}_{ij}$ are the first and second fundamental form of the mid-surface:

$$\begin{aligned}\bar{a}_{ij} &= R^{-2}\partial_i\mathbf{X}_0 \cdot \partial_j\mathbf{X}_0,\\ \bar{b}_{ij} &= R^{-3}\partial_i\partial_j\mathbf{X}_0 \cdot \partial_j\mathbf{N}_0,\\ \bar{c}_{ij} &= \bar{a}^{mn}\bar{b}_{im}\bar{b}_{jn} = \bar{b}_i^m\bar{b}_{mj},\end{aligned} \tag{17}$$

where $\mathbf{N}_0$ is the normal vector to the $\mathbf{X}_0$ mid-surface.

The coordinate expression of $\bar{a}_{ij}, \bar{b}_{ij}$ is

$$\bar{a}_{ij} = \begin{pmatrix} 1 & -\cos 2\alpha_0 \\ -\cos 2\alpha_0 & 1 \end{pmatrix}$$
$$\bar{b}_{ij} = \frac{\sin 2\alpha_0}{R} \begin{pmatrix} 0 & -1 \\ -1 & 0 \end{pmatrix} \tag{18}$$

and $\bar{c}_{ij} = R^{-2} \cos 2\alpha_0 \begin{pmatrix} 1 & 1 \\ 1 & 0 \end{pmatrix}$. The reference area element is

$$\mathrm{d}S = R^2 \sqrt{\det \bar{a}}\, \mathrm{d}\beta \mathrm{d}\theta = \frac{R^2 \sin 2\alpha_0}{2}\, \mathrm{d}\beta \mathrm{d}\theta, \tag{19}$$

which is positive for $\alpha_0 \in [0, \pi/2]$. The area element is zero at the poles of the base 2-sphere, where $2\alpha_0 = 0, \pi$. The geometric frustration manifests in the fact that $\bar{\mathbf{a}}$ and $\bar{\mathbf{b}}$ are incompatible in a surface embedded in Euclidean space because $\det \bar{\mathbf{b}} / \det \bar{\mathbf{a}} = -1/R^2$, while $K(\bar{\mathbf{a}}) = 0$, violating Gauss' *Theorema Egregium*.

We follow the Kirchhoff-Love assumption for thin sheet elasticity [3–5], namely that the shell is in a state of plane-stress and the normal direction of the mid-surface in the reference metric stay as the normal direction of the mid-surface in the actual metric. In this case, the elastic energy reduces to

$$\mathscr{E}(\alpha, \beta, \theta) = \frac{1}{8} R^3 \sqrt{\det \bar{g}_{ij}}\, \mathcal{A}^{ijkl} (g_{ij} - \bar{g}_{kl})(g_{ij} - \bar{g}_{kl}). \tag{20}$$

The elastic tensor, with indices restricted to the directions on the surface is

$$\mathcal{A}^{ijkl}(\alpha, \beta, \theta) = \frac{Y}{1 - \nu^2} \left(\nu \bar{g}^{ij} \bar{g}^{kl} + (1 - \nu) \bar{g}^{ik} \bar{g}^{jl} \right). \tag{21}$$

The total elastic energy of the shell can then be written in the form

$$E^{\text{shell}} \equiv \frac{1}{2} \int_{-\frac{W}{2R}}^{\frac{W}{2R}} \mathrm{d}\beta \int_{-\frac{L}{2R}}^{\frac{L}{2R}} \mathrm{d}\theta \sqrt{\det \bar{a}}\, \mathscr{E}_{\text{shell}}. \tag{22}$$

The elastic energy density $\mathscr{E}$, which now only depends on (β, θ), is

$$\mathscr{E}^{\text{shell}} \equiv \frac{2}{\sqrt{\det \bar{a}}} \int_{-h/2R}^{h/2R} \mathrm{d}t\, \mathscr{E}(\alpha_0 + t, \beta, \theta) \tag{23}$$

where the pre-factor in front of the integral is a convention.

Next, we expand the elastic tensor, the strain tensor and the area element in Eq. (20) to second order in t and integrate over t. Since the integration in Eq. (23) is symmetric around $t = 0$, the terms of $\mathscr{E}(\alpha_0 + t, \beta, \theta)$ linear in t integrate to zero. Thus, we are left with terms of $\mathcal{O}(1)$ and $\mathcal{O}(t^2)$ in $\mathscr{E}$, which after integration, leads to an elastic energy $E^{\text{shell}} = E^{\text{stretch}} + E^{\text{bend}}$, with

$$\begin{aligned} E^{\text{stretch}} =& \frac{Yh}{8(1-\nu^2)} \int_{\bar{M}} dA [\nu \mathrm{Tr}^2(\mathbf{a} - \bar{\mathbf{a}}) \\ &+ (1 - \nu) \mathrm{Tr}(\mathbf{a} - \bar{\mathbf{a}})^2], \\ E^{\text{bend}} =& \frac{Yh^3}{12(1-\nu^2)} \int_{\bar{M}} dA [\nu \mathrm{Tr}^2(\mathbf{b} - \bar{\mathbf{b}}) \\ &+ (1 - \nu) \mathrm{Tr}(\mathbf{b} - \bar{\mathbf{b}})^2], \end{aligned} \tag{24}$$

proving Eq. (7) in the main text.

IV. SHORT-RANGE REPULSION AND ITS CORRECTION TO BENDING ENERGY

In this section we derive the energy associated with electrostatic repulsion of the assembled sheets.

The electrostatic repulsion energy of a 2D sheet can be written as

$$E_{\text{repulsion}} = \int_{\bar{M}} \sqrt{g} d^2\sigma \rho(\sigma) \phi(\sigma) \tag{25}$$

where σ is the 2D coordinate of the sheet, $\rho(\sigma)$ and $\phi(\sigma)$ are the charge density and electric potential at σ, and g is the metric. Assuming that the surface is uniformly charged with charge Q and charge density $\rho = Q/\text{Area}[\Sigma]$, we have

$$E_{\text{repulsion}} = \frac{h^2 \rho^2}{4\pi\epsilon} \int_{\bar{M}} \sqrt{g}\, \mathrm{d}^2\sigma \int_{\bar{M}} \sqrt{g}\, \mathrm{d}^2\sigma' \frac{\exp\left(-|\mathbf{R}(\sigma) - \mathbf{R}(\sigma')|/\xi\right)}{|\mathbf{R}(\sigma) - \mathbf{R}(\sigma')|}, \tag{26}$$

where ϵ is the dielectric constant, and ξ is the screening length.

In order to simplify this repulsion energy into a local form, we can describe the sheet using the Monge parametrization, assuming the sheet is locally nearly flat. To this end, we take a plane Π external to $\bar{M}$, we choose two orthogonal coordinates (x, y) on Π, and a z direction, orthogonal to Π. The surface $\bar{M}$ is embedded as

$$\mathbf{R}(x, y) = (x, y, f(x, y)). \tag{27}$$

Using this representation in Eq. (26), expanding both the distance and the metric, and integrate over the area of the short-range repulsion, we arrive at

$$E_{\text{repulsion}} = \frac{\pi}{8}\frac{h^2\rho^2\xi^3}{4\pi\epsilon}\int_\Sigma \left[(f_{xx}+f_{yy})^2 - 4(f_{xx}f_{yy}-f_{xy}^2)\right]\sqrt{1+f_x^2+f_y^2}\,\mathrm{d}^2x_0. \tag{28}$$

The energy density in Eq. (28) from short-ranged repulsion is:

$$\mathscr{E}_{\text{repulsion}} = \frac{\pi}{8}\frac{h^2\rho^2\xi^3}{4\pi\epsilon}[(f_{xx}+f_{yy})^2 - 4(f_{xx}f_{yy}-f_{xy})^2]. \tag{29}$$

Here the first term is $(\mathrm{Tr}\mathbf{b})^2$ and the second is $\det\mathbf{b}$. Using $\mathrm{Tr}(\mathbf{b}^2) = (\mathrm{Tr}\mathbf{b})^2 - 2\det\mathbf{b}$ (valid for Monge parametrization) we rewrite

$$\mathscr{E}_{\text{repulsion}} = Q[-(\mathrm{Tr}\,\mathbf{b})^2 + 2\mathrm{Tr}(\mathbf{b}^2)], \quad Q \equiv \frac{\pi}{8}\frac{h^2\rho^2\xi^3}{4\pi\epsilon}. \tag{30}$$

The elastic part of the bending energy density is [Eqs. (24)]

$$\mathscr{E}^{\text{bend}}_{\text{elastic}} = \frac{\kappa}{2}[\nu\mathrm{Tr}^2(\mathbf{b}-\bar{\mathbf{b}}) + (1-\nu)\mathrm{Tr}(\mathbf{b}-\bar{\mathbf{b}})^2] \quad \text{where} \quad \kappa = \frac{h^3Y}{12(1-\nu^2)}. \tag{31}$$

The total energy density from elastic bending energy and repulsion energy is then

$$\mathscr{E}^{\text{bend}}_{\text{eff}} = \mathscr{E}^{\text{bend}}_{\text{elastic}} + \mathscr{E}_{\text{repulsion}} \tag{32}$$

Summing the two energy densities and using linearity of the trace on sums of matrices i.e. $\mathrm{Tr}(\mathbf{b}-\bar{\mathbf{b}})^2 = \mathrm{Tr}(\mathbf{b}^2) + \mathrm{Tr}(\bar{\mathbf{b}}^2) - 2\mathrm{Tr}(\mathbf{b}\cdot\bar{\mathbf{b}})$ etc, we find

$$\begin{aligned}\mathscr{E}^{\text{bend}}_{\text{eff}} =& \frac{1}{2}(\kappa\nu - 2Q)(\mathrm{Tr}\,\mathbf{b})^2 + \frac{1}{2}(\kappa(1-\nu)+4Q)\mathrm{Tr}(\mathbf{b}^2) \\ &- \nu\kappa(\mathrm{Tr}\mathbf{b})(\mathrm{Tr}\bar{\mathbf{b}}) - \kappa(1-\nu)\mathrm{Tr}(\mathbf{b}\cdot\bar{\mathbf{b}}) + ...\end{aligned} \tag{33}$$

where the remaining terms are constants containing the trace of $\bar{\mathbf{b}}$. The terms can be rearranged in the form of a bending energy:

$$\mathscr{E}^{\text{bend}}_{\text{eff}} = \frac{\kappa_{\text{eff}}}{2}[\nu_{\text{eff}}\mathrm{Tr}^2(\mathbf{b}-\bar{\mathbf{b}}_{\text{eff}}) + (1-\nu_{\text{eff}})\mathrm{Tr}(\mathbf{b}-\bar{\mathbf{b}}_{\text{eff}})^2], \tag{34}$$

with

$$\kappa_{\text{eff}} = \kappa + 2Q \tag{35}$$

$$\nu_{\text{eff}} = \frac{\kappa\nu - 2Q}{\kappa + 2Q} \tag{36}$$

and

$$\bar{b}_{\text{eff},xx} = \frac{\kappa(1-\nu)+2Q}{\kappa(1-\nu)+4Q}\bar{b}_{xx} + \frac{2Q}{\kappa(1-\nu)+4Q}\bar{b}_{yy} \tag{37}$$

$$\bar{b}_{\text{eff},yy} = \frac{2Q}{\kappa(1-\nu)+4Q}\bar{b}_{xx} + \frac{\kappa(1-\nu)+2Q}{\kappa(1-\nu)+4Q}\bar{b}_{yy} \tag{38}$$

$$\bar{b}_{\text{eff},xy} = \frac{\kappa(1-\nu)}{\kappa(1-\nu)+4Q}\bar{b}_{xy} \tag{39}$$

The new reference curvature satisfies $\mathrm{Tr}\,\bar{\mathbf{b}}_{\text{eff}} = \mathrm{Tr}\,\bar{\mathbf{b}}$=0. Only the traceless part of the reference curvature is changed, increasing the reference radius of curvature from the 600-cell radius R to a renormalized radius ℓR. In particular, the bare reference curvature $\bar{\mathbf{b}}$ in Eq. (6) in the main text is renormalized to

$$\bar{b}^{\text{eff}}_{ij} \equiv \frac{1}{\ell R}\bar{b}_{ij} = \frac{\kappa(1-\nu)}{\kappa(1-\nu)+4Q}\bar{b}_{ij} = \frac{1}{1+\frac{3}{2}\frac{\xi^3}{\epsilon h}\frac{\rho^2(1+\nu)}{Y}}\bar{b}_{ij} \tag{40}$$

where we used the expression of Q and κ in the last equality. The radius of the 3-sphere is thus renormalized by a factor ℓ given by

$$R \to \ell R, \quad \ell = 1 + \frac{3}{2}\frac{\xi^3}{\epsilon h}\frac{\rho^2(1+\nu)}{Y}. \tag{41}$$

The effective radius effectively increases with the charge density and the third power of the Debye screening length, thus decreasing the reference radius of curvature of the self-assembly. In the limit of $Q \gg \kappa$, this change takes the form of Eq. (15) in the main text.

In the bending-dominated regime, we have $\rho > \rho_c$ (Eq. (16) in the main text). Substituting in Eq. (41), we find $\ell > 1 + W^2/\phi a h$. Using $W \sim 500\,nm$, $a \sim 5\,nm$ and $5 < h < 100\,nm$, we find $\ell > 340 \gg 1$. This estimate uses the intrinsic curvature radius of the reference state. If we use the extrinsic curvature radius of the final morphology, as it is done in thin shell theory, we find $\ell \sim 500 \gg 1$ as discussed in the main text.

V. MORPHOLOGY OF THE RIBBON IN THE BENDING DOMINATED LIMIT

In this section we study the solutions to the equilibrium problem $\delta E = 0$ where the energy is the sum of the elastic and the repulsion parts, $E = E_{\text{eff}} + E_{\text{repulsion}}$. We take the assumption that $E_{\text{eff}}^{\text{bend}} = 0$ so $\delta E = \delta E^{\text{stretch}}$. This is accurate in the limit $W \ll \sqrt{\ell R h}$ where R is the repulsion corrected radius of curvature of the reference metric, h is the thickness, and ℓ is the dimensionless constant characterizing the ratio between the repulsion and the elasticity strength (defined in the main text). Because of the repulsion corrections, this limit is satisfied for the ribbons assembled in the experiment.

A. Solving for the actual metric and the morphology

As mentioned in the main text, we take the mid-surface of the shell on the Clifford torus at $\alpha = \pi/4$. The reference first and second fundamental forms of the shell are Eq. (18) evaluated at $\alpha = \pi/4$ and with the correction from repulsion:

$$\bar{\mathbf{a}}(\beta,\theta) = \begin{pmatrix} 1 & 0 \\ 0 & 1 \end{pmatrix}, \quad \bar{\mathbf{b}}_{\mathbf{eff}}(\beta,\theta) = \frac{1}{\ell R}\begin{pmatrix} 0 & -1 \\ -1 & 0 \end{pmatrix}. \tag{42}$$

In the discussions below we will drop the "eff" for convenience. The coordinate θ runs along great circles circumscribed to right-handed tetrahelices. As we discussed in the main text, we take the actual metric $\mathbf{a}$ to be translationally invariant along θ direction, which is the direction along these tetrahelices. The metric Ansatz is then a function of β:

$$\mathbf{a}(\beta) = \begin{pmatrix} E(\beta) & F(\beta) \\ F(\beta) & G(\beta) \end{pmatrix}. \tag{43}$$

In the bending-dominated limit, the bending energy vanishes iff $\mathbf{b} = \bar{\mathbf{b}}$, where $\bar{\mathbf{b}}$ is written in Eq. (42). Then, $\mathbf{a}(\beta)$ is the 1st fundamental form of a surface $\mathbf{R}(\beta,\theta)$ with normal vector $\mathbf{N}$ and second fundamental form $\mathbf{N}\cdot\partial_i\partial_j\mathbf{R} = \bar{b}_{ij}$ iff it satisfies the Gaussi-Codazzi-Peterson-Mainardi (GCPM) equations [6]

$$\partial_2\bar{b}_{11} - \partial_1\bar{b}_{12} = \bar{b}_{11}\Gamma^1_{12} + \bar{b}_{11}(\Gamma^2_{12} - \Gamma^1_{11}) - \Gamma^2_{11}\bar{b}_{22} \tag{44a}$$

$$\partial_2\bar{b}_{21} - \partial_1\bar{b}_{22} = \bar{b}_{11}\Gamma^1_{22} + \bar{b}_{11}(\Gamma^2_{22} - \Gamma^1_{12}) - \Gamma^2_{12}\bar{b}_{22} \tag{44b}$$

and the Gauss equation

$$\frac{\det\bar{\mathbf{b}}}{\det\mathbf{a}} = -\frac{1}{E}(\partial_2\Gamma^2_{12} - \partial_1\Gamma^2_{11} + \Gamma^1_{12}\Gamma^2_{11} + \Gamma^2_{12}\Gamma^2_{12} - \Gamma^2_{11}\Gamma^2_{22} - \Gamma^1_{11}\Gamma^2_{12}) \tag{45}$$

where Γ^i_{jk} are the Christoffell symbols:

$$\Gamma^k_{ij} = \frac{1}{2}a^{km}(\partial_i a_{mj} + \partial_j a_{mi} - \partial_m a_{ij}), \tag{46}$$

and where we defined $x^1 = \beta$ and $x^2 = \theta$ to keep the notation simple. The explicit expressions of the Christoffel symbols are:

$$\Gamma^1_{11} = \frac{GE_{,\beta} - 2FF_{,\beta}}{2\det a} \qquad \Gamma^2_{11} = -\frac{FE_{,\beta} - 2EF_{,\beta}}{2\det a} \tag{47a}$$

$$\Gamma^1_{12} = \frac{FG_{,\beta}}{2\det a} \qquad \Gamma^2_{12} = -\frac{EG_{,\beta}}{2\det a} \tag{47b}$$

$$\Gamma^1_{22} = \frac{GG_{,\beta}}{2\det a} \qquad \Gamma^2_{22} = -\frac{FG_{,\beta}}{2\det a} \tag{47c}$$

where we indicated partial differentiation with a comma symbol. Using the expression of $\bar{\mathbf{b}}$ and Eq. (47) in Eq. (44), the GCPM equations reduce to

$$\frac{FE_{,\beta}}{F^2 - EG} = 0 \tag{48}$$

$$\frac{EG_{,\beta} - GE_{,\beta} - 2FF_{,\beta}}{F^2 - EG} = 0. \tag{49}$$

From the first equation, we either have $E(\beta) = E_0 = \text{const}$ or $F = 0$. In the former case, we cannot satisfy the second equation so the only non-trivial solution is $F = 0$, leading to a diagonal metric. Then, from the second equation we find $EG_{,\beta} = GE_{,\beta}$, i.e.

$$E(\beta) = cG(\beta), \;\; c = \text{const} \quad . \tag{50}$$

Using Eq. (50), the expression of $\bar{\mathbf{b}}$ and $\det \mathbf{a} = EG - F^2$ in Eq. (45) the Gauss equation reduces to a differential equation for G:

$$c\,GG_{,\beta\beta} - 2G - c\,GG_{,\beta} = 0, \tag{51}$$

with solution

$$G(\beta) = \frac{c_1}{c_2^4}\left[\exp\left(\frac{c_1\beta}{2}\right) + \frac{c_1^2}{c_2}\exp\left(-\frac{c_1\beta}{2}\right)\right]. \tag{52}$$

Using this result in Eq. (43) the Ansatz reduces to

$$\mathbf{a}(\beta) = \frac{c_1}{c_2^4}\left[\exp\left(\frac{c_1\beta}{2}\right) + \frac{c_1^2}{c_2}\exp\left(-\frac{c_1\beta}{2}\right)\right]\begin{pmatrix} c & 0 \\ 0 & 1 \end{pmatrix}. \tag{53}$$

The determinant of $\mathbf{a}$ is $\det \mathbf{a} = c\,c_1^{-8}c_2^{-2}e^{-2c_1\beta}(c_1^2 + c_2 e^{c_1\beta})^4$, so we find that $c > 0$.

The stretching energy density of the final ansatz Eq. (53) can be computed from the elastic strain tensor:

$$\epsilon_{ij} = \frac{1}{2}(a_{ij}(\beta) - \delta_{ij}). \tag{54}$$

Since β is the width direction, we suppose W small compared to $\sqrt{Rh}$ and expand the stretching energy density to order β^5. Then, we integrate over the width to find the total stretching energy $E_{\text{stretch}}(c, c_1, c_2)$ that depends parmetrically on the integration constants c, c_1, c_2. The expressions are long and not illuminating. The condition of stationarity of $E_{\text{stretch}}(c, c_1, c_2)$ reduces to 2 equations in 3 unknowns:

$$(c_1^2 + c_2)[(1 + c^2 + 2c\nu)(c_2^2 + 2c_1^2c_2) + c_1^4(1 + c^2 - c(c_2(1+\nu) - 2\nu) - c_2(2+\nu))] = 0 \tag{55a}$$

$$(c_1^2 + c_2)[2c_1^2c_2(c+\nu) + c_2^2(c+\nu) + c_1^4(c + \nu - c_2(1+\nu))] = 0 \tag{55b}$$

One solution is $c_2 = -c_1^2$ for all c. The other is

$$c = 1 \quad , \quad c_2 = \frac{c_1^2}{2}(c_1^2 - 2 - c_1\sqrt{c_1^2 - 4}) \quad . \tag{56}$$

Using Eq. (56) in Eq. (53), we can compute the stress

$$\sigma_{ij} = 2\mu\,\epsilon_{ij} + \lambda\epsilon_k^k\,\delta_{ij} \tag{57}$$

and impose a vanishing stress at the boundary (free BC) $\sigma_{11}(W) = 0$ to fix the parameter c_1. The result is

$$c_1 = -\sqrt{2}\sqrt{1 + \frac{1}{W^2} - \frac{\sqrt{1-2W^2}}{W^2}} \quad . \tag{58}$$

Since $W/\sqrt{\ell R h}$ is small, we formally expand $\mathbf{a}$ in W and keep only the lowest order:

$$\mathbf{a}(\beta) = \begin{pmatrix} \cosh^2\beta & 0 \\ 0 & \cosh^2\beta \end{pmatrix} + o(W/R) \tag{59}$$

where the linear correction is $-\cosh\beta\sinh\beta\delta_{ij}$. We could solve the Weingarten equations to find the surface corresponding to the actual metric Eq. (59) and the off-diagonal 2nd fundamental form in Eq. (42). More simply, we observe that the mean curvature vanishes ($H \sim \mathrm{Tr}(\bar{\mathbf{b}}) = 0$) so the solution must be a minimal surface. Moreover, the second fundamental form is off-diagonal, so the surface has pure torsion and zero bending: it is a ruled surface generated by the rotation of a straight line around a curve. Finally, the Gaussian curvature of the surface is negative: $\det\bar{\mathbf{b}}/\det\mathbf{a} = -1/(\ell^2R^2\cosh^4\beta)$. The solution is an helicoid with embedding

$$\mathbf{R}(\beta,\theta) = \ell R(\sinh\theta\cos\beta, \sinh\theta\sin\beta, \beta). \tag{60}$$

The solution is a Right-handed helicoid. We can check that the first fundamental form $a_{ij} = \partial_i\mathbf{R}\cdot\partial_j\mathbf{R}$ is Eq. (59). The second fundamental form of the helicoid is $b_{ij} = \mathbf{N}\cdot\partial_i\partial_j\mathbf{R}$. By explicit calculation of the unit normal vector

$$\mathbf{N} = \frac{\partial_\beta\mathbf{R}\times\partial_\theta\mathbf{N}}{|\partial_\beta\mathbf{R}\times\partial_\theta\mathbf{N}|}, \tag{61}$$

we can verify that $\mathbf{b} = \bar{\mathbf{b}}$. Notice that the positive sign in front of the third component of the embedding $R^3 = \beta$ in Eq. (60) determines the handedness of the solution. To summarize:

- We start from a crystal of right-handed tetrahelices, which are aligned with the θ direction of the coordinates
- The actual metric is taken to be translationally invariant along θ, the long direction of the ribbons, due to the chiral symmetry breaking of the tetrahelices self-assembly pathway and energy minimization in the limit of $L \gg W$
- We minimize the elastic energy in the bending-dominated limit with free boundary conditions, finding an helicoid of the same handedness as the tetrahelices.

B. Tetrahelix handedness determines the helicoid's handedness

In the previous subsection we studied how *right*-handed tetrahelices may self-assemble into a *right*-handed thin shell. We can analyze a thin shell of *left*-handed tetrahelices by interchanging β with θ. The reference metric $\bar{\mathbf{g}}$ (and therefore $\bar{\mathbf{a}}$ and $\bar{\mathbf{b}}$) is invariant under this transformation. Let us call S_R the right-handed helicoid and S_L the helicoid obtained by exchanging β and θ. Let ($\mathbf{a}_R$, $\mathbf{b}_R$) and ($\mathbf{a}_L$, $\mathbf{b}_L$) be their respective 1st and 2nd fundamental forms. If the normal to S_R is $\mathbf{N}_R$ (see Eq. (61)) then the normal to S_L is $-\mathbf{N}_R$. The reason is that the order of the tangent vectors $\partial_\beta\mathbf{R}$ and $\partial_\theta\mathbf{R}$ is exchanged under $\theta \leftrightarrow \beta$. Consequently, the 2nd fundamental form of S_L ($b^L_{ij} = \mathbf{N}_L\cdot\partial_i\partial_j\mathbf{R}_L$) is $-\mathbf{b}_R$. The solution must satisfy $\mathbf{b}_L = \bar{\mathbf{b}}_L$. But $\bar{\mathbf{b}}_L = \bar{\mathbf{b}}_R$. In order to compensate for the minus sign, we have to reflect the coordinates of $\mathbb{E}^3$, e.g. by reflecting $R^3 \to -R^3$, so the embedding is a Left-handed helicoid:

$$\mathbf{R}_L(\beta,\theta) = \ell R(\sinh\beta\cos\theta, \sinh\beta\sin\theta, -\theta). \tag{62}$$

This predicts that tetrahelices and self-assembled thin shells must have the same handedness. The right- and left-handed solutions have the same energy. The symmetry is broken by the chiral ligands: right- (left-) handed ligands can induce the formation of enantiopure right- (left-) handed tetrahelices, and eventually select the chirality of the thin shell. This corresponence between chiralities is indeed observed in the experiments. Notice that we defined the handedness of the helicoid with respect to its long axis. The helicoid twists with the opposite sense of rotation around a short axis (perpendicular to the length direction).

[1] J. F. Sadoc, "Helices and helix packings derived from the {3, 3, 5} polytope," The European Physical Journal E **5**, 575–582 (2001).

[2] L.D. Landau, E.M. Lifshitz, A.M. Kosevich, J.B. Sykes, L.P. Pitaevskii, and W.H. Reid, *Theory of Elasticity*, Course of theoretical physics, Vol. 7 (Elsevier Science , 1986).

[3] Augustus Edward Hough Love, *A treatise on the mathematical theory of elasticity* (Cambridge university press, 2013).

[4] Philippe G Ciarlet, "An introduction to differential geometry with applications to elasticity," Journal of Elasticity **78**, 1–215 (2005).

[5] Efi Efrati, Eran Sharon, and Raz Kupferman, "Elastic theory of unconstrained non-euclidean plates," Journal of the Mechanics and Physics of Solids **57**, 762–775 (2009).

[6] Manfredo P Do Carmo, *Differential geometry of curves and surfaces: revised and updated second edition* (Courier Dover Publications, 2016).
